# ASSOCIATIONS OF MEAN COGNITIVE PERFORMANCE AND INTRAINDIVIDUAL VARIABILITY WITH WHITE MATTER LESIONS

**DOI:** 10.1093/geroni/igae098.3806

**Published:** 2024-12-31

**Authors:** Uriel Urias, Kelsey Thomas, Mary Ellen Garcia, Amanda Gonzalez, Britney Luu, Victoria Gutierrez, Katherine Bangen

**Affiliations:** San Diego State University, San Diego, California, United States; University of California San Diego, La Jolla, California, United States; University of California, San Diego, San Diego, California, United States; University of California, San Diego, San Diego, California, United States; San Diego State University, San Diego, California, United States; San Diego State University, San Diego, California, United States; University of California, San Diego, La Jolla, California, United States

## Abstract

White matter lesions (WMLs) are magnetic resonance imaging markers of small vessel cerebrovascular disease and have been associated with Alzheimer’s disease and related dementias. Intraindividual variability (IIV), often measured as variability in performance across neuropsychological tests at a single time point, has emerged as a sensitive marker of early cognitive difficulties that may have utility beyond measures of mean neuropsychological performance (MP). However, the associations of IIV with neuroimaging variables remains understudied. This study included 70 older adults without dementia (mean age=68.57) who completed a comprehensive neuropsychological battery and underwent MRI brain scans. WMLs were adjusted for estimated total intracranial volume and transformed to reduce skew. Linear regression models examined the associations of IIV, MP, and WMLs both across and within the cognitive domains of memory, attention/processing speed, and executive function. Covariates included age, sex, and education. IIV showed no significant association with WMLs, neither within nor across the domains examined (all p’s>.05). In the domains of memory (B=-.035, p=.025), executive function (B=-.039, p=.018), and in global cognition (B=-.065, p=.003) lower MP was associated with more WMLs. In the attention/processing speed domain, MP was not significantly associated with WMLs (all p’s >.05). The present study only examined cross-sectional data, so future studies should re-evaluate the associations with IIV, MP, and WMLs longitudinally. While the small sample size may limit the generalizability of this study, these findings suggest that measures of MP may better reflect WMLs relative to measures of IIV.

